# The feasibility and validity of a preference-weighted composite endpoint to establish value in geriatric care

**DOI:** 10.1186/1472-6963-14-S2-P55

**Published:** 2014-07-07

**Authors:** Cynthia Hofman, Jennifer Lutomski, Han Boter, Bianca Buurman, Ton De Craen, Marcel OldeRikkert, Rogier Donders, Peter Makai, René Melis

**Affiliations:** 1 Geriatric Medicine, Radboud University Medical Center, Nijmegen, The Netherlands; 2 Health Evidence, Radboud University Medical Center, Nijmegen, The Netherlands; 3 Epidemiology, University Medical Centre Groningen, Groningen, The Netherlands; 4 Internal Medicine and Geriatrics, Academic Medical Center, Amsterdam, The Netherlands; 5 Gerontology and Geriatrics, Leiden University Medical Centre, Leiden, The Netherlands

## Background

As part of the Dutch National Care for the Elderly Programme, The Older Persons and Informal Caregivers Survey Minimum Data Set (TOPICS-MDS) was developed to gather uniform information on outcome measures. Furthermore, to combine the outcome measures into one single index and to promote comparability between studies, a preference-weighted Composite End Point (called: TOPICS-CEP) was developed [1]. The aim of this study was to validate TOPICS-CEP in a large heterogeneous sample of older persons aged ≥65 years.

## Materials and methods

Data from 17,603 older persons were derived from TOPICS-MDS (http://www.TOPICS-MDS.eu); a public data repository. Feasibility was evaluated by the prevalence of missing values among TOPICS-CEP scores. To assess convergent validity, TOPICS-CEP scores were cross validated against the Cantril’s ladder life satisfaction scale and the EuroQol-5D utility score. Known-group validity of TOPICS-CEP was investigated across socio-demographic and clinical characteristics. To assess whether TOPICS-CEP scores were generalizeble across different settings, we conducted pooled and subgroup analyses: older persons in the general population, general practitioner setting, and hospital.

## Results

In the complete sample, TOPICS-CEP scores could be calculated for the majority of the participants (88.7%). There were no floor and ceiling effects found and the distribution was slightly skewed to the left. The correlation between TOPICS-CEP and Cantril’s ladder was 0.43 (95%CI [0.39-0.48]) and the correlation between TOPICS-CEP and EuroQol-5D was 0.63 (95%CI [0.58-0.67]). Expectedly, mean TOPICS-CEP scores differed significantly (p<0.05) across marital status (married or cohabiting: 7.50 versus partner deceased: 7.13), living arrangements (independent living with others: 7.56 versus dependent living: 6.37), dementia (no: 7.43 versus yes: 6.30), depression (no: 7.42 versus yes: 6.26), and dizziness with falls (no: 749 versus yes: 6.42). When stratified by subgroups, similar results were found for feasibility, convergent and known-group validity.

## Conclusions

The TOPICS-CEP was able to accurately reflect general wellbeing in a large pooled dataset as well as across subgroups. Our data support that the TOPICS-CEP score is an objective and robust measure for researchers interested in investigating the general well-being of older persons. The TOPICS-CEP guideline version 1.1 is now available online http://www.TOPICS-MDS.eu.
